# AAV delivered lysosome-targeting chimeras mediate sustained antibody depletion in vivo

**DOI:** 10.64898/2026.07.05.736665

**Published:** 2026-11-03

**Authors:** Jonathan Lee Yang, Kang Yong Loh, Cindy Rosaura Sandoval Espinoza, Dina Schuster, Sean A. Yamada-Hunter, Louai Labanieh, Elena Sotillo, Crystal Mackall, Karl Deisseroth, Carolyn Bertozzi

## Abstract

Immunoglobulin G (IgG) is a critical effector of the adaptive immune system, but IgG autoantibodies against self-antigens drive pathology across a wide range of autoimmune diseases. Lowering circulating IgG is a validated and promising therapeutic avenue. Here we developed genetically-encoded lysosome targeting chimeras (GELYTACs) that target circulating IgGs for clearance and degradation. The GELYTACs comprised two protein modules derived from insulin-like growth factor 2 (IGF2) and an IgG-binding nanobody, respectively, and mediated clearance of plasma IgG via the lysosomal trafficking receptor IGF2R. To achieve long-lasting IgG depletion, we encoded GELYTACs in an AAV gene therapy vector and established continuous expression in mice. We also developed conditional GELYACs that are activatable with disease-specific proteases or small molecule drugs and show that GELYTACs can be secreted from cellular therapies. This work establishes GELYTACs as a possible therapeutic modality that is deliverable using genetic medicine approaches.

## Full Text Availability

The license terms selected by the author(s) for this preprint version do not permit archiving in PMC. The full text is available from the preprint server.

